# Supplementary material for: Pleiotropy promotes the evolution of inducible immune responses in a model of host-pathogen coevolution
Source: PLoS Comput Biol. 2023 Apr 6;19(4):e1010445. doi: 10.1371/journal.pcbi.1010445 (PMC10079112; doi:10.1371/journal.pcbi.1010445)
Supplement: S1 Text — Table A. Names, values, and description for variables and parameters used in the simulation. Fig B. Diagram of infection period, end states of infection, and example of how key findings were drawn from infection data. A) the host is infected, and the infection dynamics are calculated as described in the Methods Simulation Framework step 4. Infection ends in one of 3 ways: B) the parasite is managed, but not killed before the 20 timesteps have passed, C) the parasite is killed before the 20 step limit is reached, D) the parasite goes unmanaged and kills the host after 20 time steps have passed. E) shows an example of how the data used to generate immune response density plots were collected. Following the conclusion of the infection, the difference between initial effector abundance and maximum effector abundance was determined for each infected host. The percent of the maximal abundance that was induced by parasites was then calculated for each host and used to generate an immune response probability density plot for the population. Created with BioRender.com. Fig C. Pleiotropic hosts can outcompete non-pleiotropic hosts. Results of competition simulations. Rows correspond to infection percentages and columns correspond to the pleiotropy type for a set of competitions. Unevolved competitions are those that had non-pleiotropic and pleiotropic organism enter competition immediately. Evolved are those that took place after 250 generations of adaptation in isolated populations. Fig D-I. Winners of competition simulations are consistently more inducible than corresponding losers, but pleiotropic and non-pleiotropic hosts are similarly inducible when matched for winning and losing. The immune response density plots of winners and losers of competitive simulations after 250 generations of adaptation. Comparisons presented are a) pleiotropic winners vs. non-pleiotropic losers, b) pleiotropic winners vs. pleiotropic losers, c) non-pleiotropic winners vs. pleiotropic losers, [file pcbi.1010445.s001.docx]

**Supplemental Tables and Figures for:**

Pleiotropy promotes the evolution of inducible immune responses in a model of host-pathogen coevolution

Reese A. Martin and Ann T. Tate

**Table A:** Names, values, and description for variables and parameters used in the simulation

| Variable | Value | Description |
| --- | --- | --- |
| Parasite damage (v) | 2 | The amount of damage incurred by hosts due to parasite infection, akin to virulence. Chosen so that excess immune activity and parasite burden had the same degree of effect on fitness |
| Area | [0,1] | The normalized area under the curve of parasite infection, determines host damage and parasite success in the next generation |
| [P_i_^*^] | [0,1] | The active portion of protein P_i_ |
| ${[P}_{i}]$ | [0,1] | The inactive portion of protein P_i_ |
| $k_{i,j}$ | [-1,1] | The upregulatory action of protein P_j_ on protein P_i_ |
| $I_{i,j}$ | [-1,1] | The inhibitory action of protein P_j_ on protein P_i_ |
| DeathCoef | .3 | The percentage of each population that died in a single generation |
| DeathThreshold | .9 |  |
| UseCoef | .01 | The limit of parasite burden that hosts can tolerate before being unable to breed |
| Host Mutation Rate | 5e-3 | The amount of protein P_i_ that is deactivated in a time step due to acting on protein P_j_ |
| Parasite Mutation Rate | 1e-2 | Selected to be balance scarcity of mutations found in nature against needing to run simulations for more generations. Picked in coordination with parasite mutation rate so that parasites mutate at 2x the host rate |
| ProtCost | [0,Inf) | The cost hosts pay for having a given number of proteins in their networks. Cost is zero up until 10 proteins and then increases per extra protein. |

**Figure B:** Diagram of infection period, where A) the host is infected, and the infection dynamics are calculated as described in the Methods *Simulation Framework* step 4. Infection ends in one of 3 ways: B) the parasite is managed, but not killed before the 20 timesteps have passed (outcome A in methods section 4), C) the parasite is killed before the 20 step limit is reached (outcome B in methods section 4), D) the parasite goes unmanaged and kills the host after 20 time steps have passed. E) shows an example of how the data used to generate immune response density plots were collected. Following the conclusion of the infection, the difference between initial effector abundance and maximum effector abundance was determined for each infected host. The percent of the maximal abundance that was induced by parasites was then calculated for each host and used to generate an immune response probability density plot for the population.


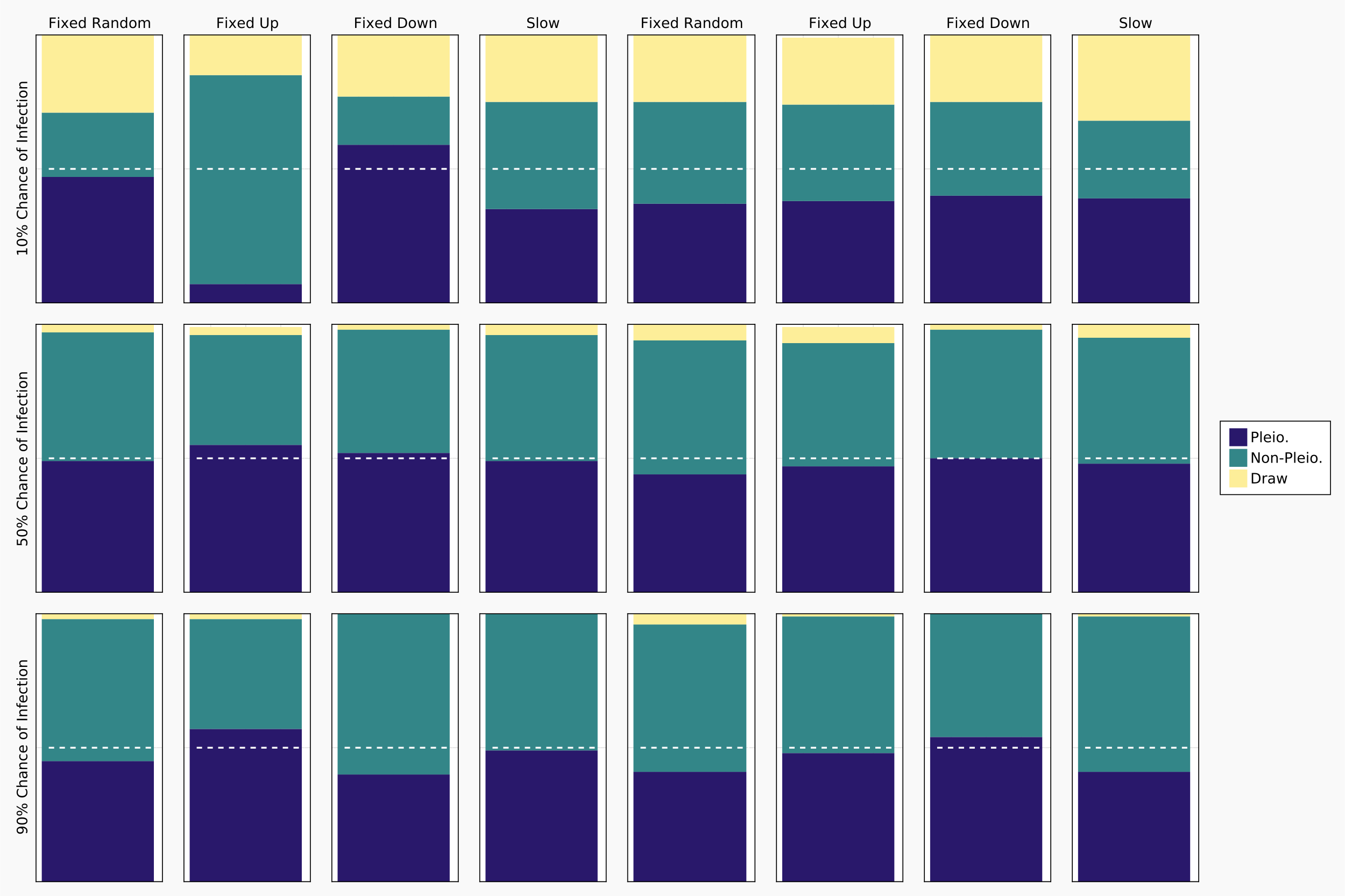


**Figure C:** Pleiotropic hosts can outcompete non-pleiotropic hosts. Results of competition simulations. Rows correspond to infection percentages and columns correspond to the pleiotropy type for a set of competitions. The size of each bar corresponds to the number of simulations the indicated host type won the competition. Unevolved competitions are those that had non-pleiotropic and pleiotropic organism enter competition immediately. Evolved are those that took place after 250 generations of adaptation in isolated populations.


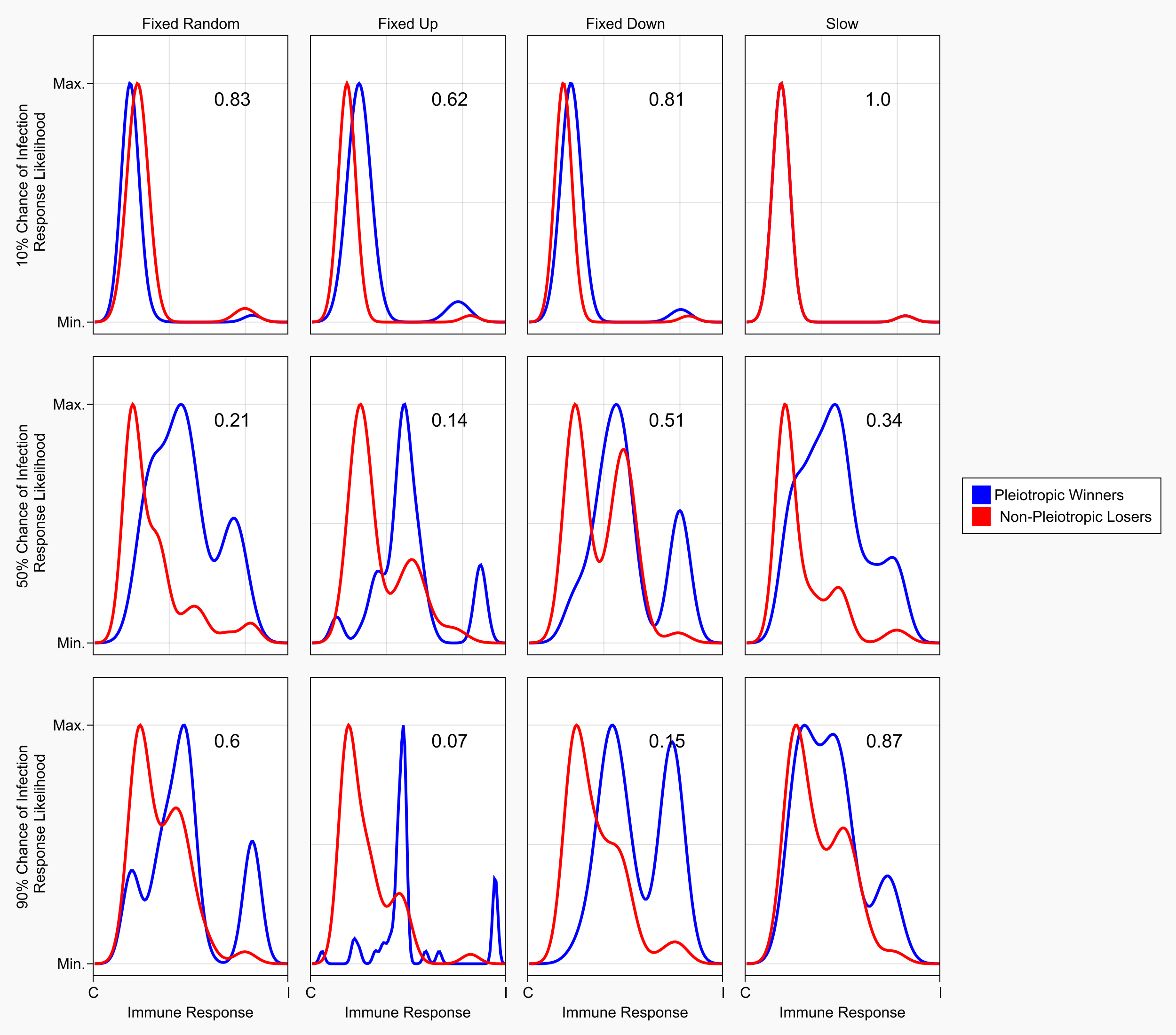


**Figure D:** Winners and losers of competitive simulations after 250 generations of adaptation: Pleiotropic winners (blue) vs Non-pleiotropic losers (red). The x-axis shows the percent of the response that is induced by parasites, with the left-hand side being 0% of response induced, to 100% induced responses on the right. The y-axis corresponds to the relative likelihood of finding an immune response in the specified population that is X% induced.


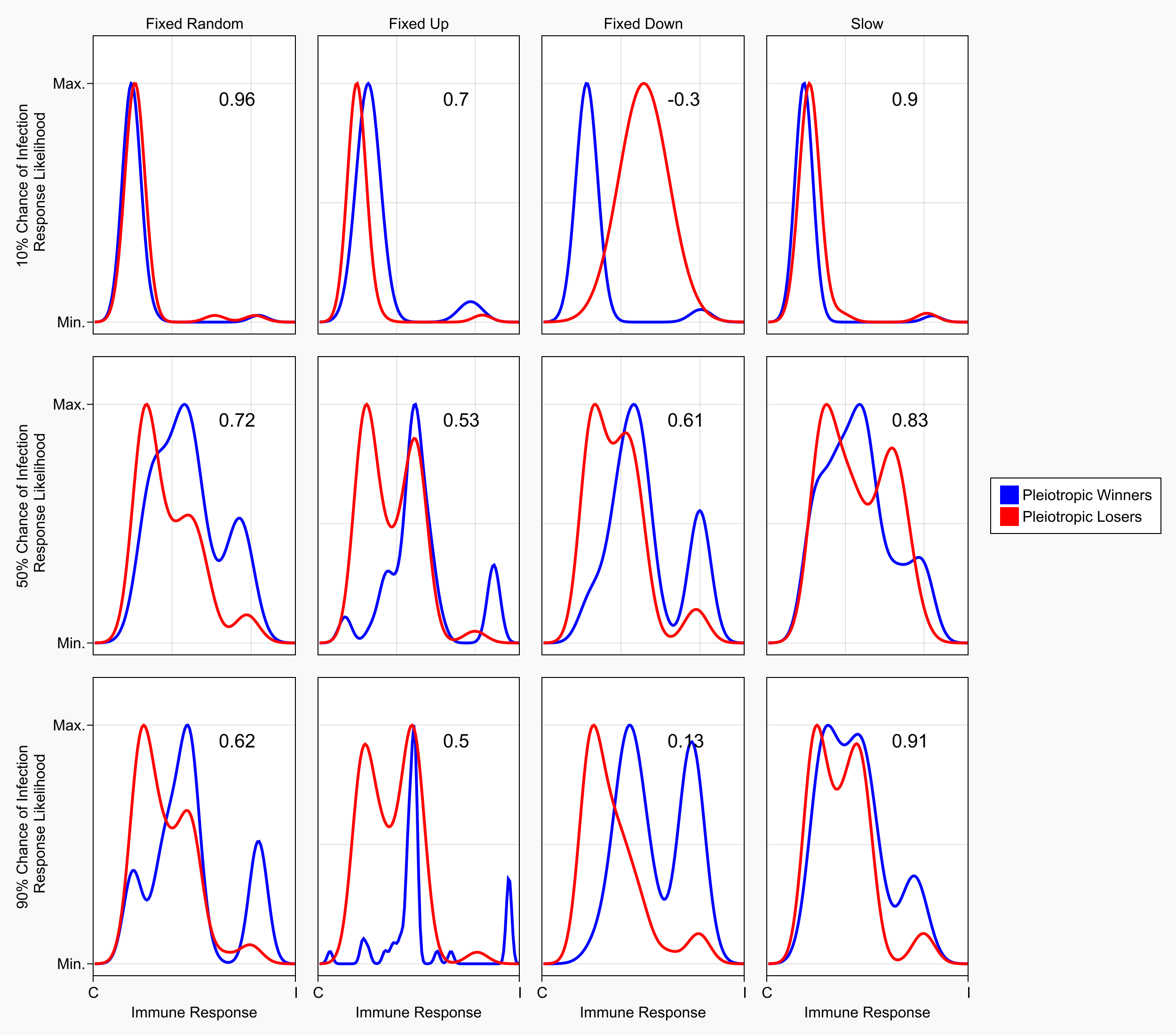


**Figure E:** Winners and losers of competitive simulations after 250 generations of adaptation**:** Pleiotropic winners (blue) vs Pleiotropic losers (red). The x-axis shows the percent of the response that is induced by parasites, with the left-hand side being 0% of response induced, to 100% induced responses on the right. The y-axis corresponds to the relative likelihood of finding an immune response in the specified population that is X% induced.


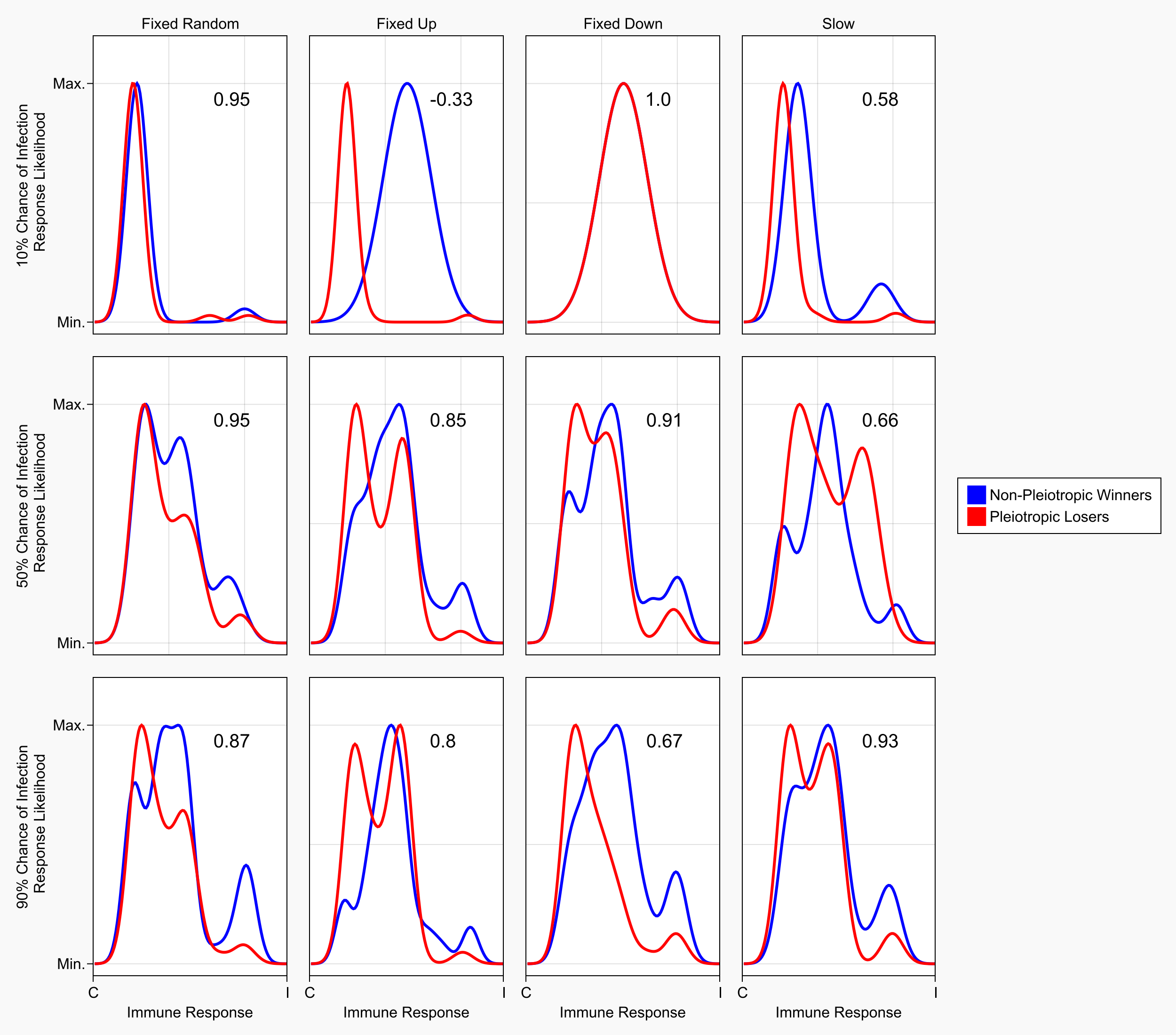


**Figure F:** Winners and losers of competitive simulations after 250 generations of adaptation**:** Non-pleiotropic winners (blue) vs Pleiotropic losers (red). The x-axis shows the percent of the response that is induced by parasites, with the left-hand side being 0% of response induced, to 100% induced responses on the right. The y-axis corresponds to the relative likelihood of finding an immune response in the specified population that is X% induced.


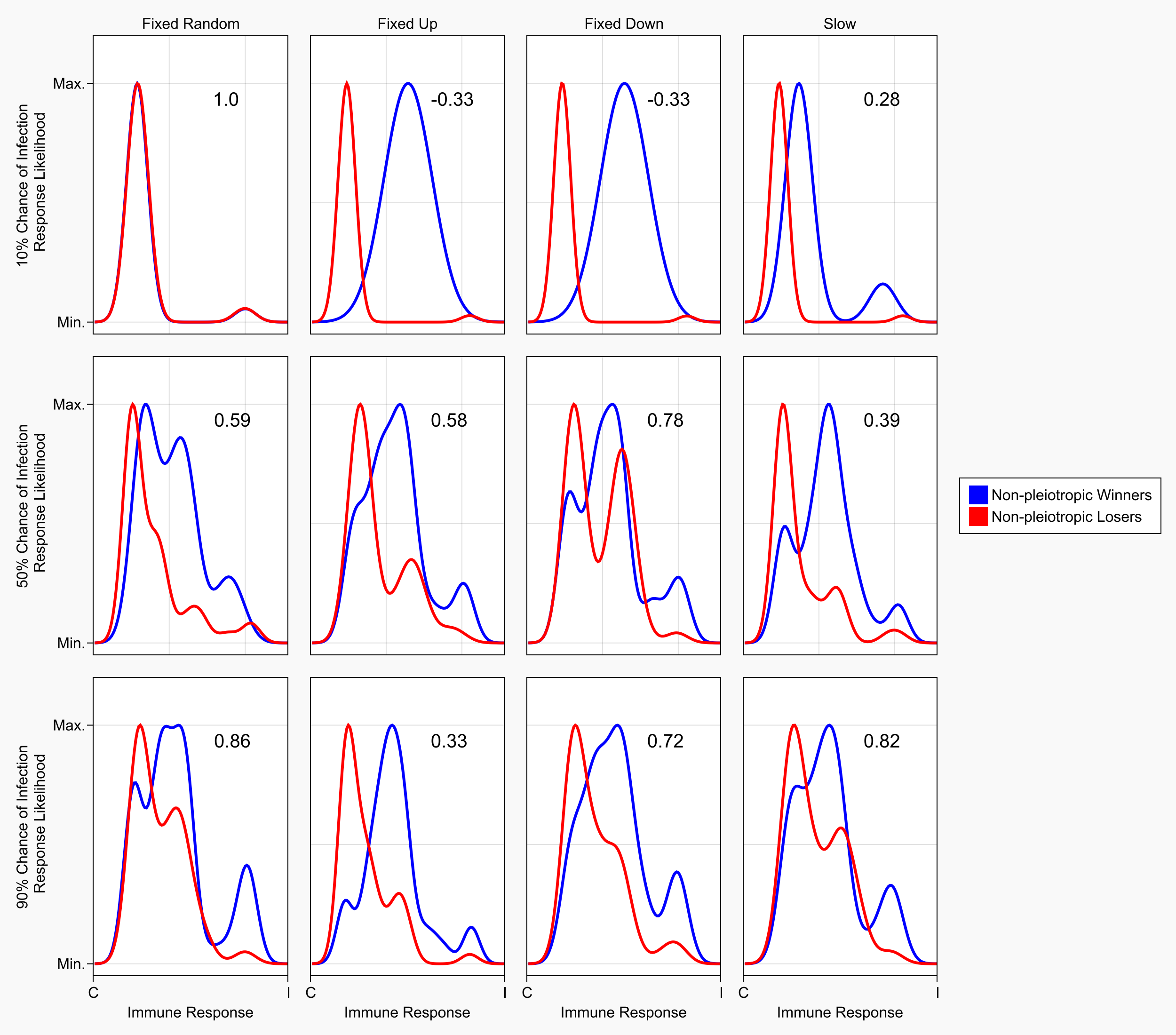


**Figure G:** Winners and losers of competitive simulations after 250 generations of adaptation**:** Non-pleiotropic winners (blue) vs Non-pleiotropic losers (red). The x-axis shows the percent of the response that is induced by parasites, with the left-hand side being 0% of response induced, to 100% induced responses on the right. The y-axis corresponds to the relative likelihood of finding an immune response in the specified population that is X% induced.


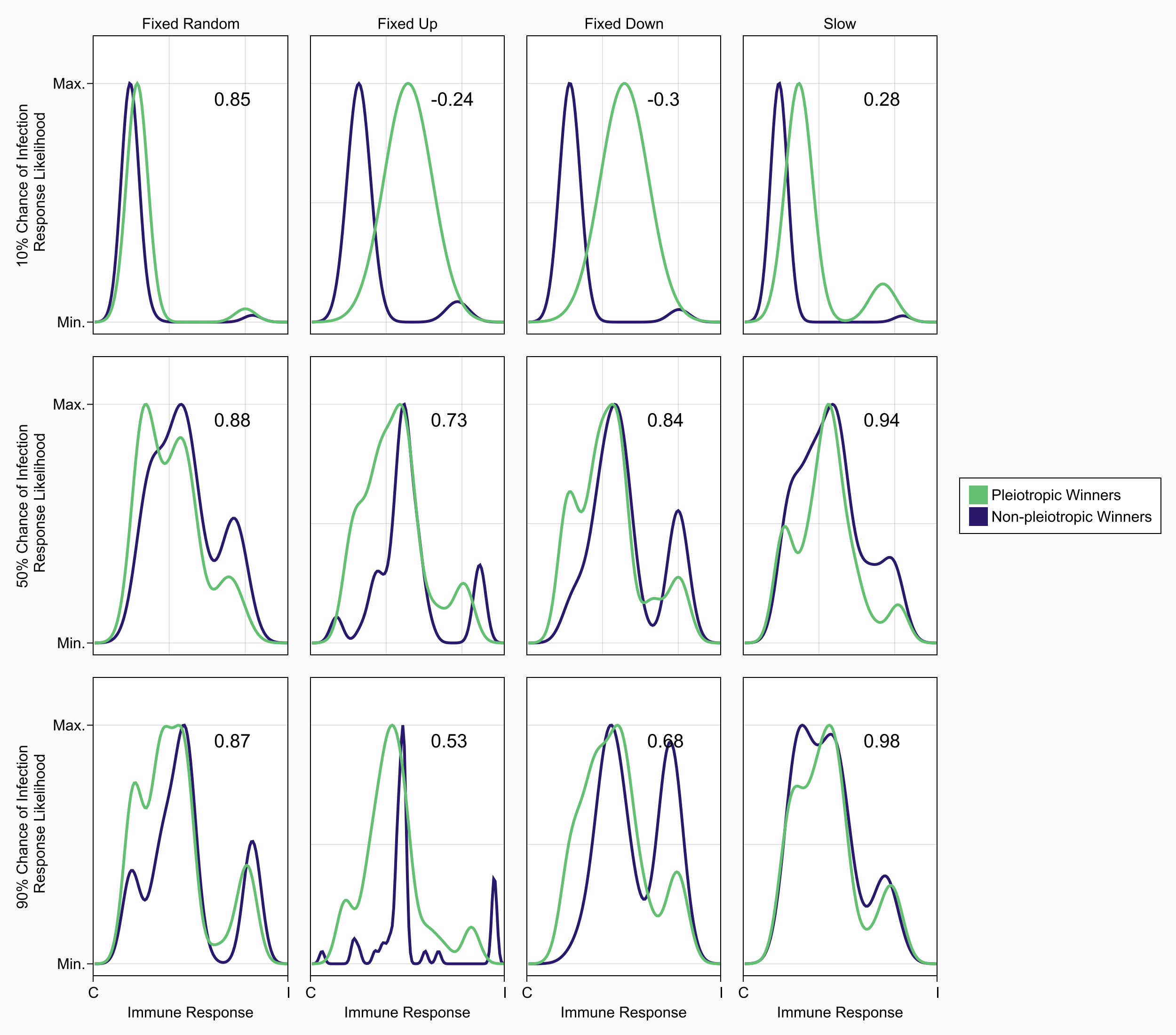


**Figure H:** Winners and losers of competitive simulations after 250 generations of adaptation: Pleiotropic winners (blue) vs Non-pleiotropic winners (green). The x-axis shows the percent of the response that is induced by parasites, with the left-hand side being 0% of response induced, to 100% induced responses on the right. The y-axis corresponds to the relative likelihood of finding an immune response in the specified population that is X% induced.


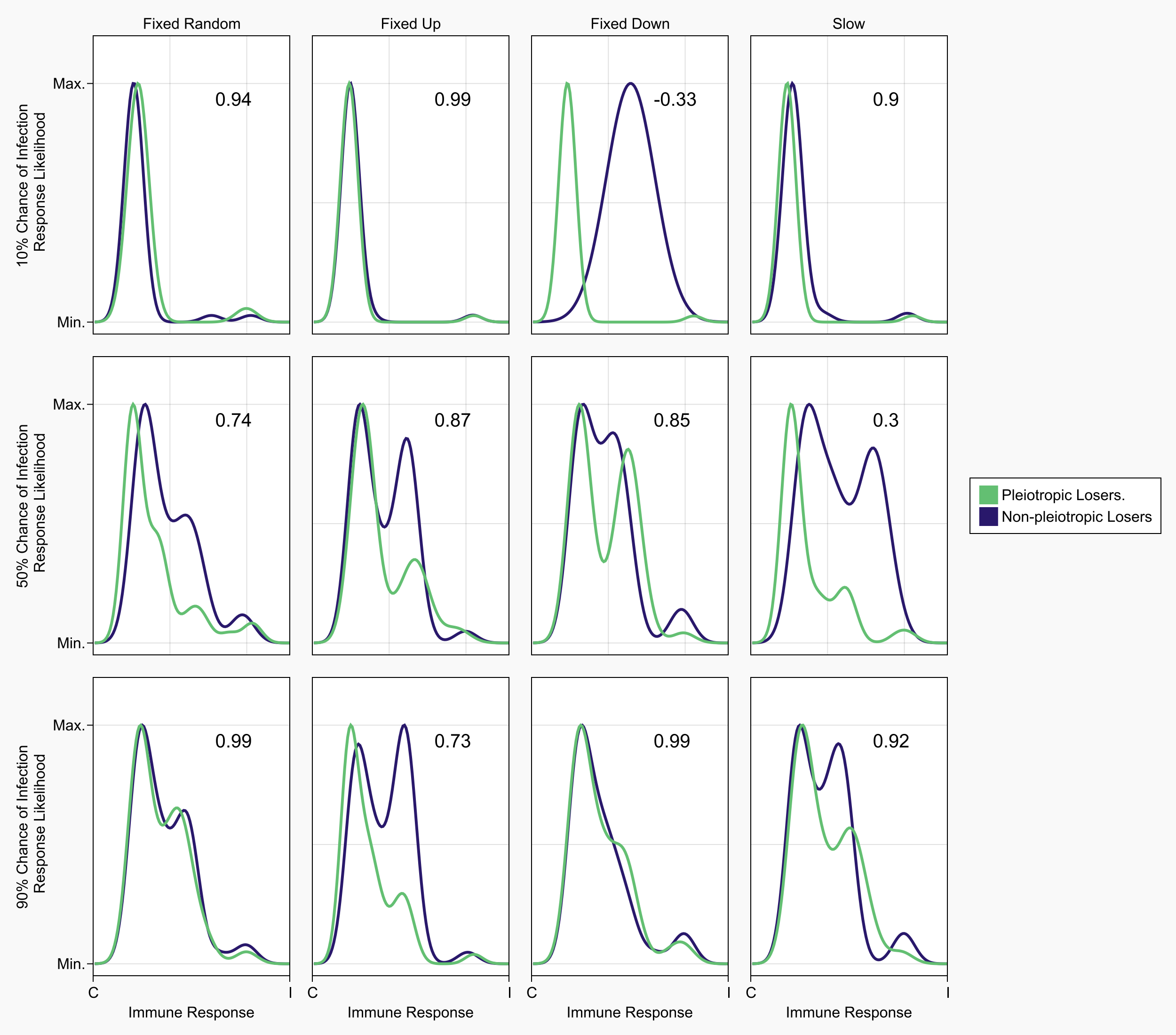


**Figure I:** Winners and losers of competitive simulations after 250 generations of adaptation**:** Pleiotropic losers (blue) vs Non-Pleiotropic losers (green). The x-axis shows the percent of the response that is induced by parasites, with the left-hand side being 0% of response induced, to 100% induced responses on the right. The y-axis corresponds to the relative likelihood of finding an immune response in the specified population that is X% induced.


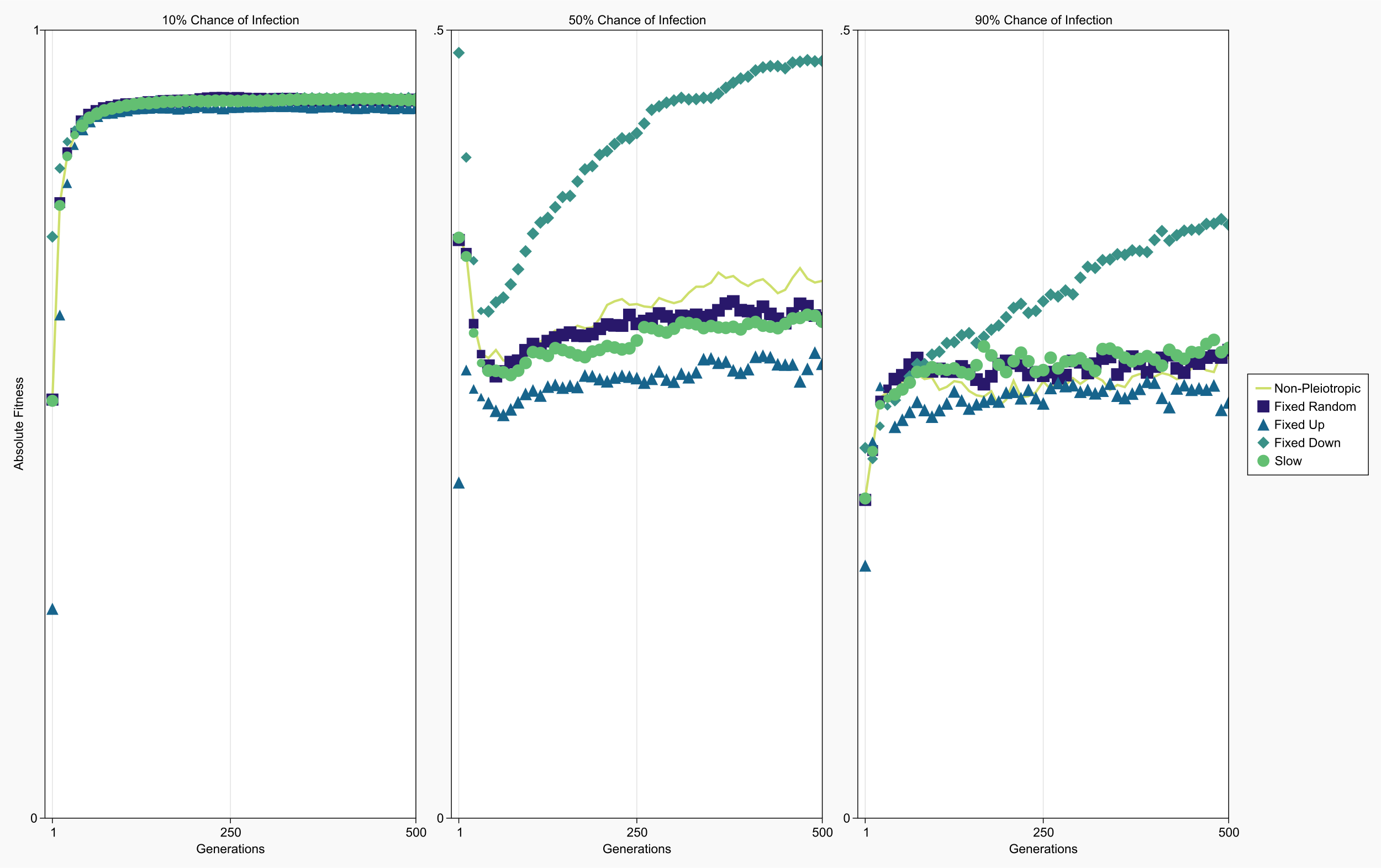


**Figure J:** Down regulatory pleiotropy results in hosts for whom fitness that equals or exceeds non-pleiotropic hosts. Plots of average host fitness through 500 generations when the chance of infection was 10% (left), 50% (middle), or 90% (right). Each panel shows host or parasite fitness from unconstrained (solid line), Fixed Random (squares), Fixed Up (triangles), Fixed Down (diamonds), and 100x slower evolution (circles) simulations. Average host fitness was calculated using hosts that were and were not infected for each generation. Note that the y axis changes scale in the second and third panel because overall host fitness decreased as the chance of infection increased.


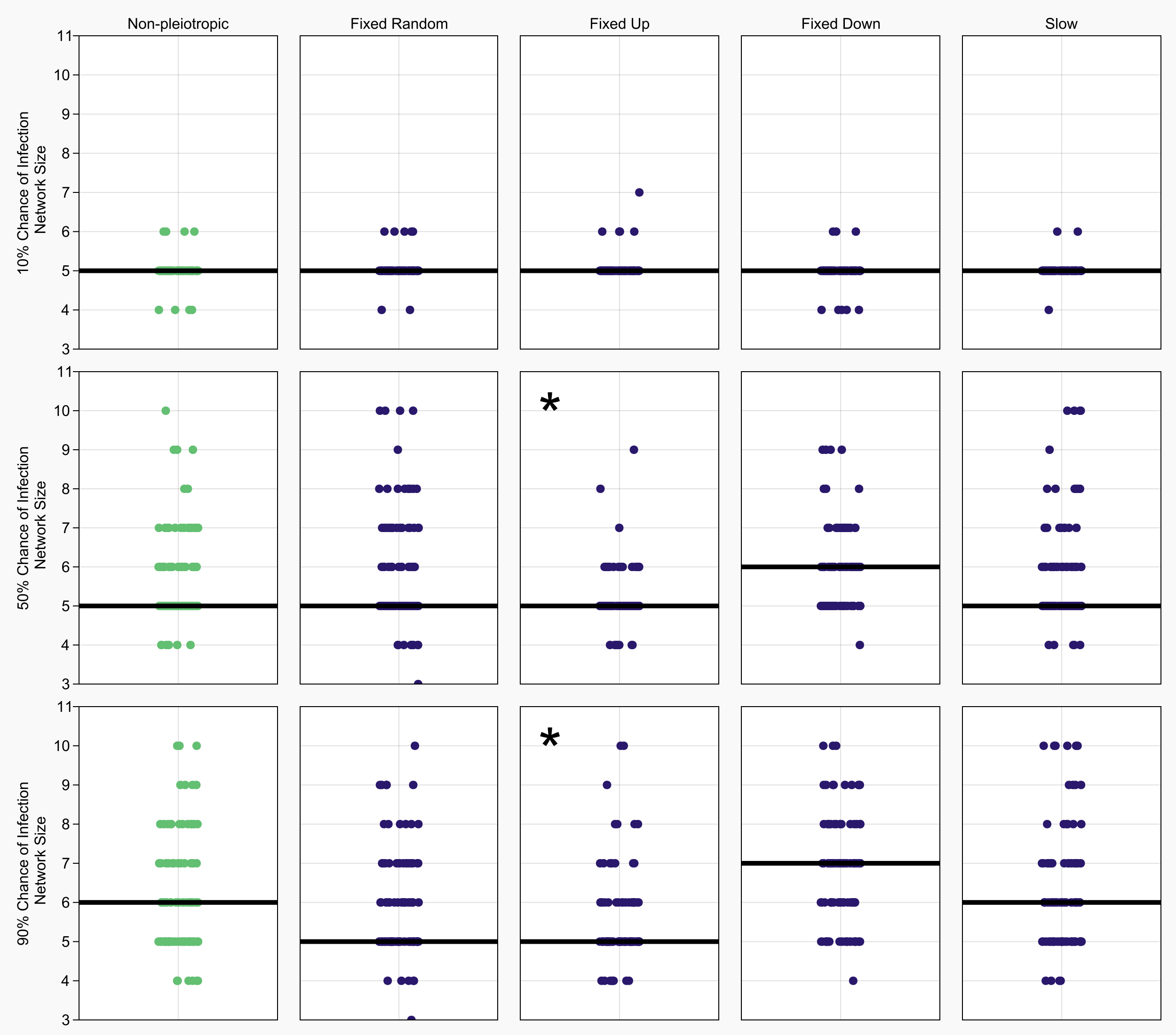


**Figure K:** Pleiotropy can significantly alter the size of host signaling networks. Size (number of proteins) of the most common network from each run of a scenario with median lines presented in black. All networks start with 5 proteins, and at the 10% infection rate, no pleiotropic conditions diverge significantly from this point. At the 50% infection level, only the fixed downregulation pleiotropic condition has an increased median network size. Conversely, at the 90% infection level, only fixed upregulation did not increase in median network size. Asterisks indicate a significant difference from the non-pleiotropic scenario in each row.


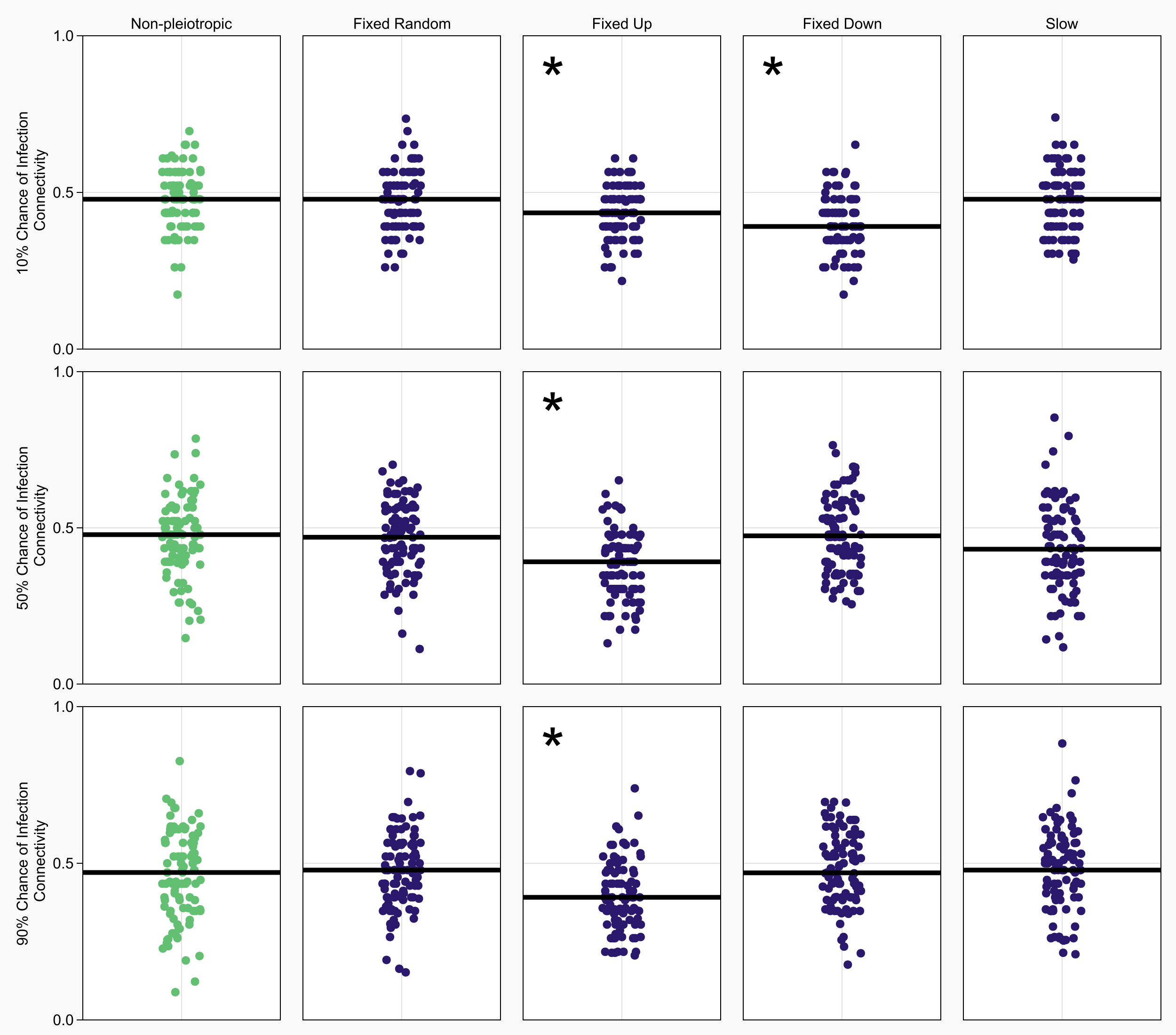


**Figure L:** Pleiotropy can significantly reduce signaling network connectivity. Network connectivity measured as the percentage of total potential connections an evolved network used. Dots represent the connectivity of the most common network at the end of each simulation. Each row is an infection risk (10, 50, or 90%) and each column is a type of pleiotropy. On average, half of all connections are used initially (mean initial connectivity is .5) . Asterisks indicate a significant difference from the non-pleiotropic scenario in each row.


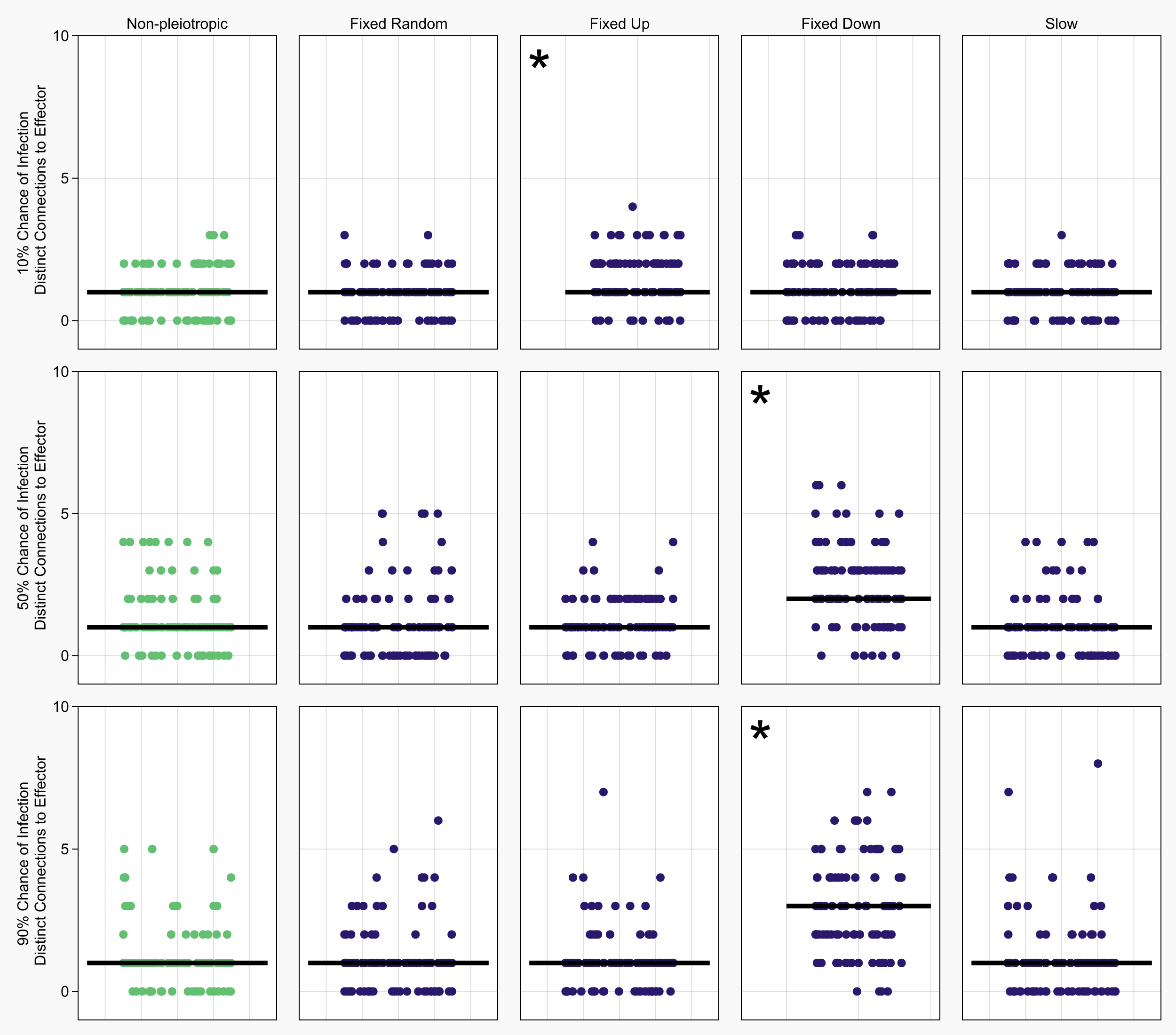


**Figure M:** Pleiotropic hosts can develop significantly more distinct paths through a network than non-pleiotropic hosts. Number of distinct paths from the detector to the effector where the collection of distinct paths in a network are the ones that have no mutual signaling proteins. Each dot is the number of distinct paths in the most common network of a simulation. The fixed downregulation conditions deploy a higher number of distinct paths. Importantly, distinct paths connect the detector to the effector in a manner that is partially insulated from other paths through the network, increasing robustness. Asterisks indicate a significant difference from the non-pleiotropic scenario in each row.


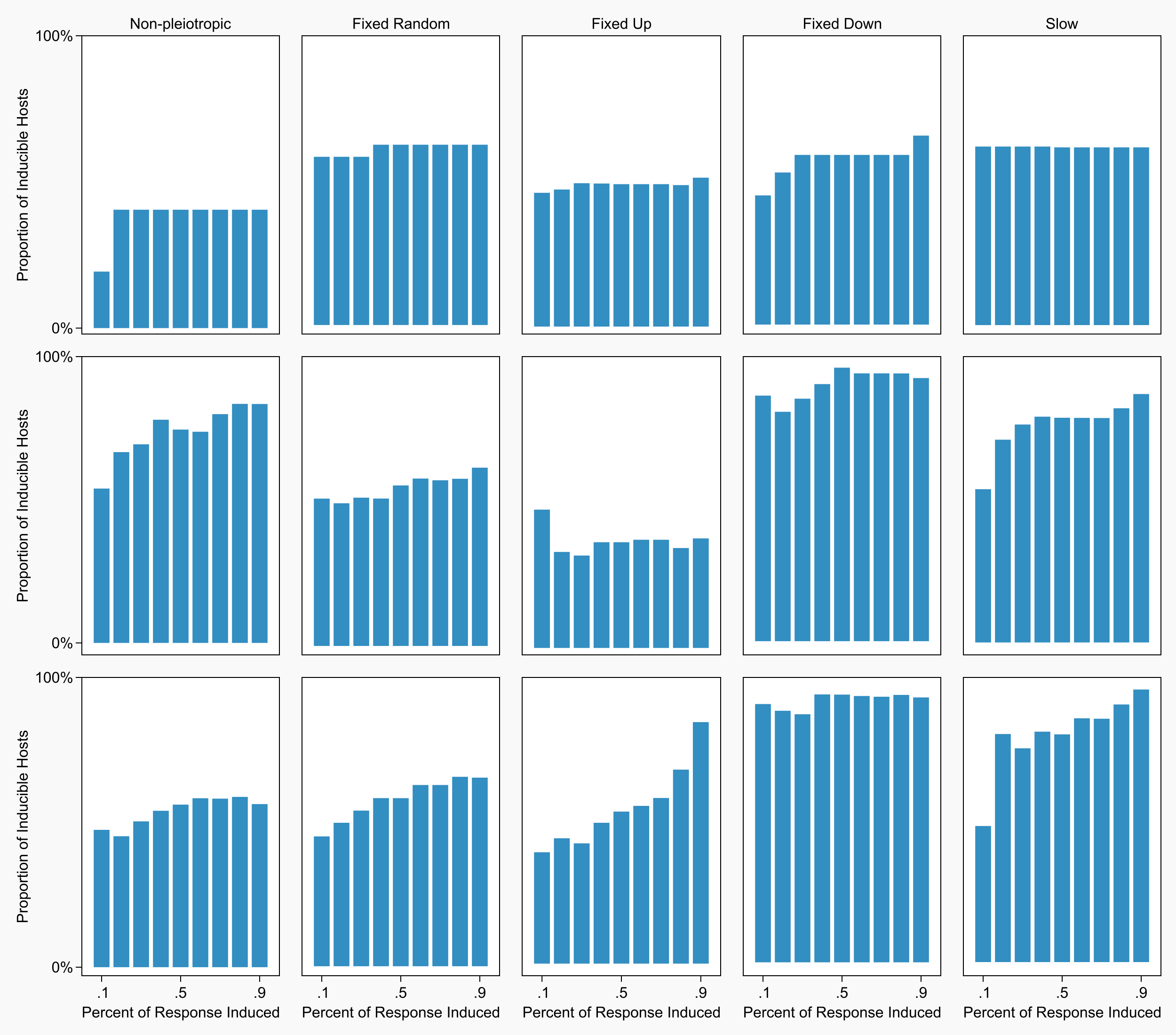


**Figure N:** A single highly inducible host in a population is often indicative of many hosts that are at least that inducible. Plots show the average percentage of a population that is at least as inducible as the level indicated on the x-axis. X-axis is the inducibility threshold and the Y-axis is the average proportion of the population that meets or exceeds that threshold given that at least one host meets or exceeds it. Columns correspond to the implementation of pleiotropy, rows correspond to the percent of the population that is infected in each generation (10, 50, 90 percent) Example from the Fixed Down column, 90% infection row: Looking at the bar labeled .9, this indicates that in all simulations with at least one host where >= 90% of the maximal immune response was induced by parasites a significant majority of hosts (~90%) where also expressing immune responses that were >= 90% inducible.


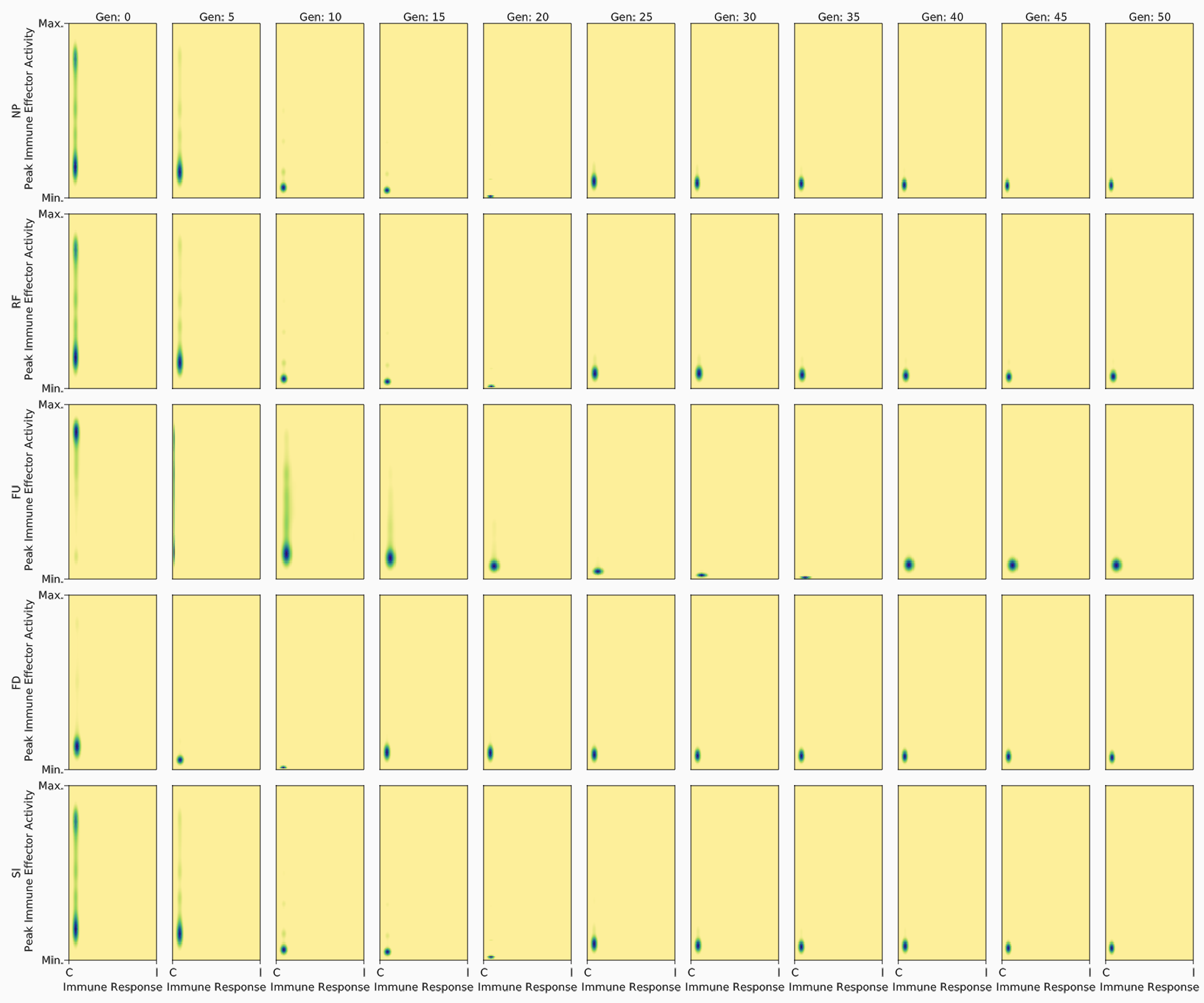


**Figure O:** When the chance of infection is low, the evolutionary trajectory of hosts does not depend on their pleiotropic status. Magnitude of immune response by the proportion of response that is induced in the initial 50 generations of an evolutionary simulation where the chance of infection was 10%. Darker colors indicate more common combinations of magnitude of immune responses and proportion of response induced by parasites. The y-axis shows the peak of immune effector activity achieved during infection range [0,1]. The x-axis shows the proportion of the peak response that was generated following infection range [0,1]


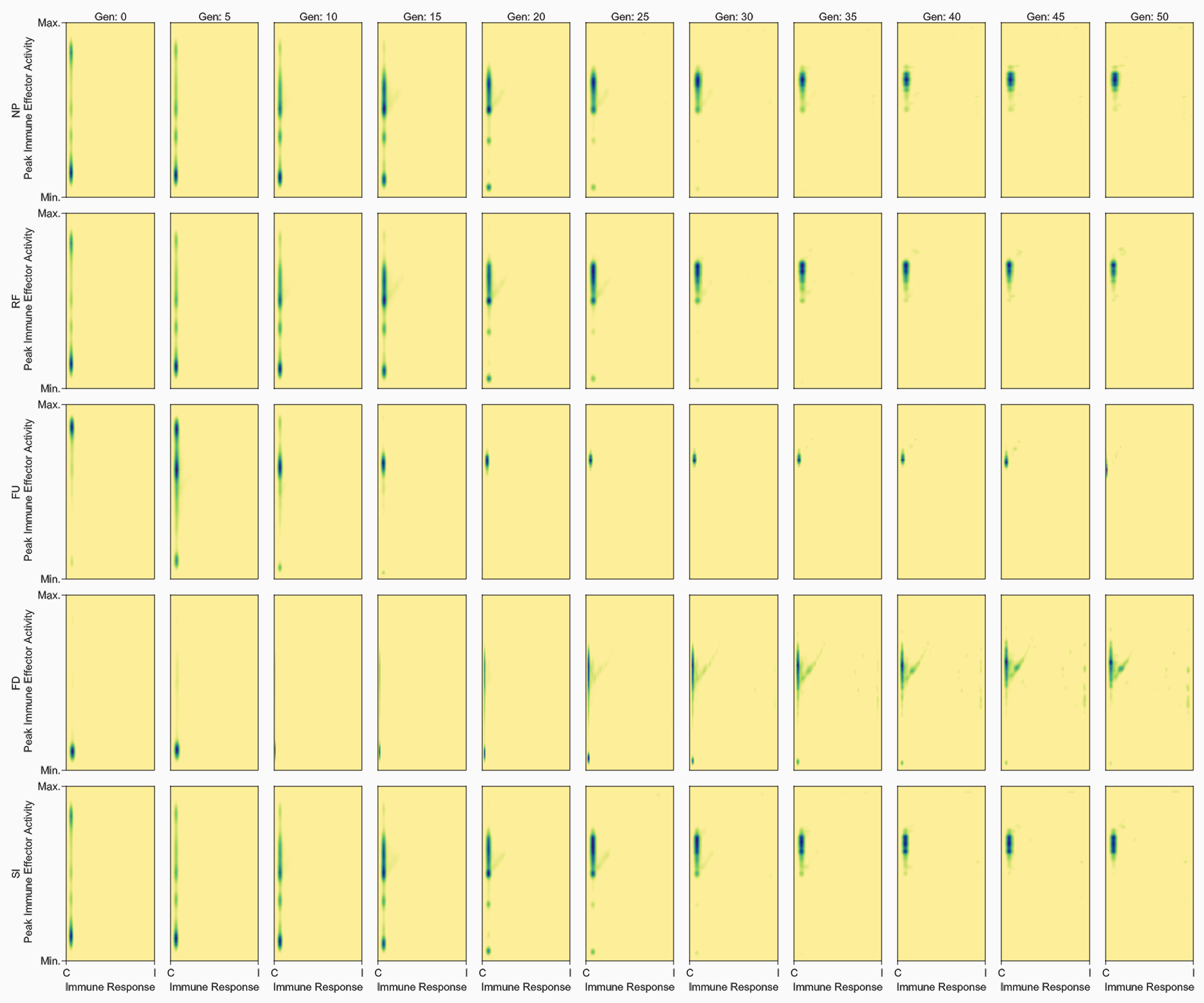


**Figure P:** When the chance of infection is moderate, downregulatory pleiotropy leads hosts to novel evolutionary trajectories. Magnitude of immune response by the proportion of response that is induced in the initial 50 generations of an evolutionary simulation where the chance of infection was 50%. Darker colors indicate more common combinations of magnitude of immune responses and proportion of response induced by parasites. The y-axis shows the peak of immune effector activity achieved during infection range [0,1]. The x-axis shows the proportion of the peak response that was generated following infection range [0,1]


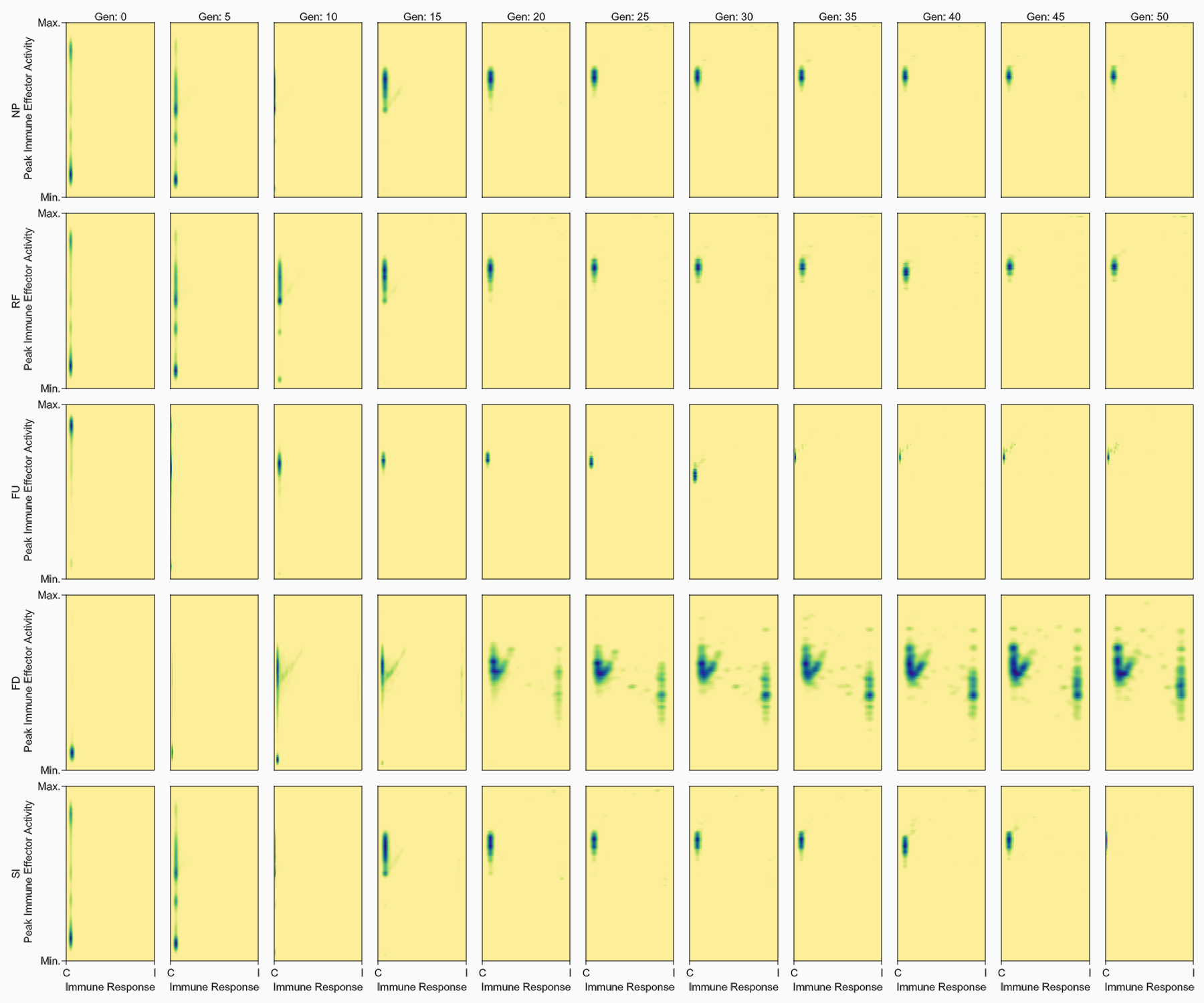


**Figure Q:** When the chance of infection is high, downregulatory and upregulatory pleiotropy led hosts to novel evolutionary trajectories. Magnitude of immune response by the proportion of response that is induced in the initial 50 generations of an evolutionary simulation where the chance of infection was 90%. Darker colors indicate more common combinations of magnitude of immune responses and proportion of response induced by parasites. The y-axis shows the peak of immune effector activity achieved during infection range [0,1]. The x-axis shows the proportion of the peak response that was generated following infection range [0,1]


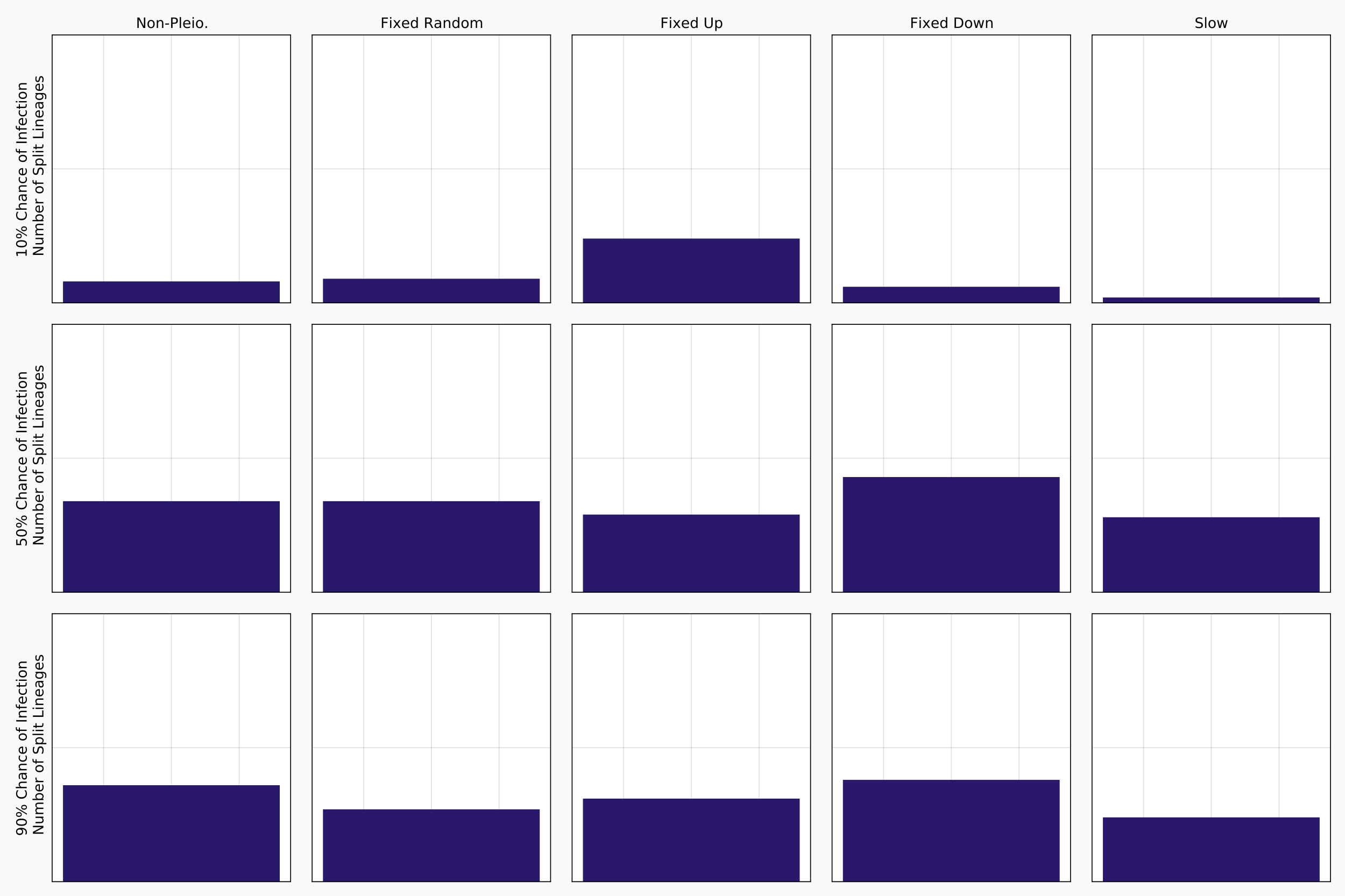


**Figure R:** As the chance of infection increases, the proportion of host lineages that contain constitutive and inducible hosts increases. The proportion of runs where hosts that descended from the same initial host ended up with immune systems with different response dynamics (i.e. a split or bifurcated lineage) in the final generation of the simulation. We refer to hosts that share an ancestor but do not share immune response dynamics as being a part of a split lineage.


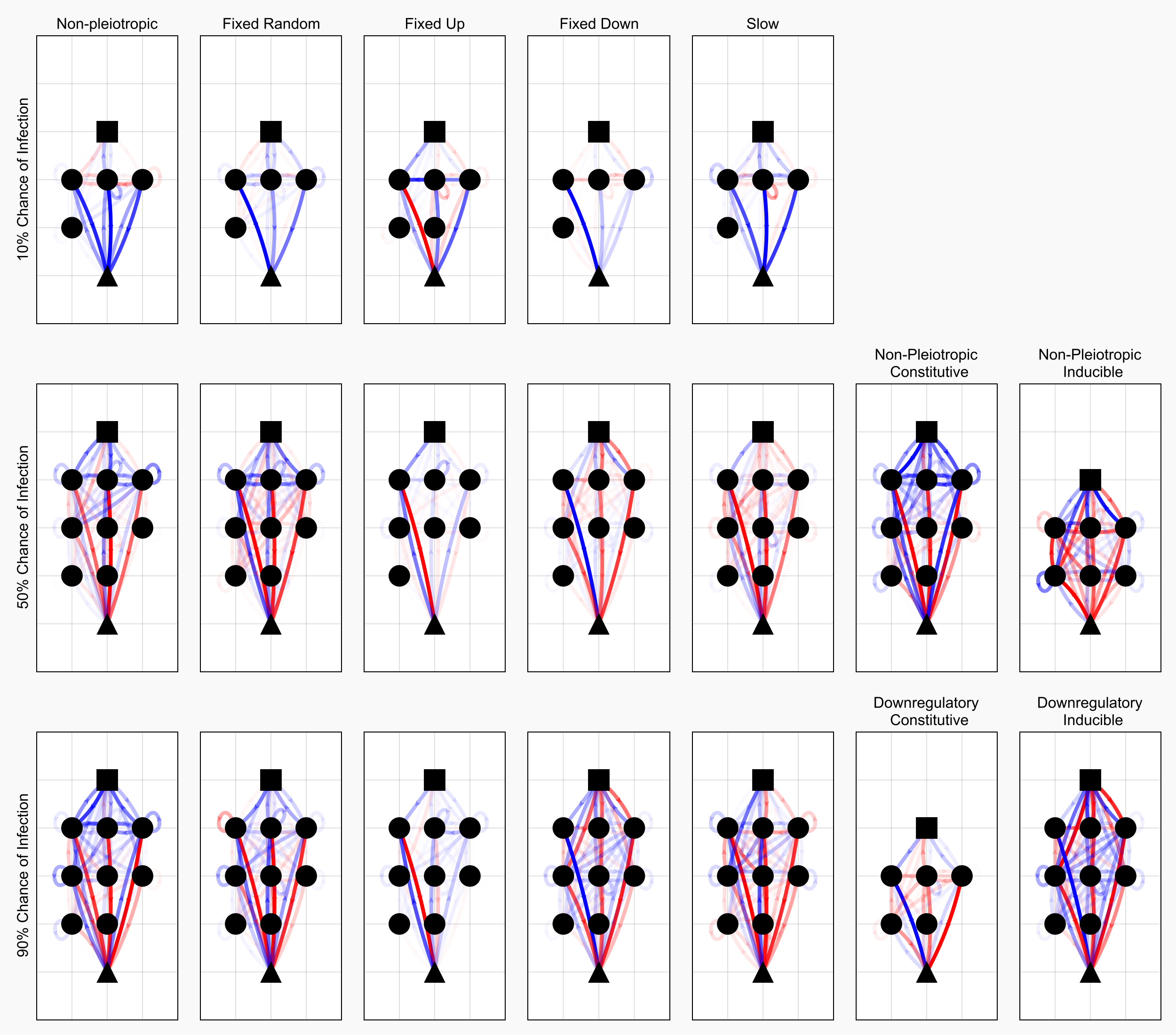


**Figure S:** The pleiotropic nature of a host shapes end state signaling networks. The average host network generated in each pleiotropic constraint and infection level pairing. The average host network generated in each pleiotropic constraint and infection level pairing. These average networks were generated using the most common networks from the end of each simulation at a given pairing. Saturation for the connections between proteins is scaled based on the most common connection across all networks at the given constraint and infection level. Arrows denote the direction of the connection, blue connections are down regulatory, red are upregulatory.
